# Health and Well-Being Consequences for Gender Violence Survivors from Isolating Gender Violence

**DOI:** 10.3390/ijerph18168626

**Published:** 2021-08-15

**Authors:** Adriana Aubert, Ramon Flecha

**Affiliations:** Department of Sociology, University of Barcelona, 08034 Barcelona, Spain; ramon.flecha@ub.edu

**Keywords:** isolating gender violence, gender-based violence, health consequences, survivors, sexual harassment, sexual abuse, bystander intervention, well-being, practitioners, policymakers

## Abstract

Recent scientific literature has published about the Isolating Gender Violence (IGV), the violence exerted by harassers against those who support their victims. IGV provokes suffering to advocates with health and well-being consequences that have been analyzed by more recent research; but IGV provokes also suffering on the victims of gender violence when they see the suffering of those who have supported them and also for their isolation. Thus, the aim of the present study is to explore the health and well-being consequences of IGV on gender violence survivors. The methodology includes three narratives of gender violence survivors whose advocates supporting them were victimized by IGV. The results show, on the one hand, an increase of the health and well-being effects of gender violence already analyzed by scientific literature; on the other hand, new health and well-being effects appear. All survivors interviewed say that, besides those new consequences for their health, the support of those advocates has decreased the global health effects of the total gender violence they suffered.

## 1. Introduction

Gender Violence is a global public health problem, according to data by WHO [1], 1 in 3 women have suffered physical and/or sexual violence worldwide. Gender Violence is defined by the United Nations as “physical, sexual or psychological harm or suffering to women, including threats of such acts, coercion or arbitrary deprivation of liberty, whether occurring in public or in private life” [2]. Regarding perpetrators, research has shown that they belong to a Dominant Traditional Masculinity [3], characterized by being violent and aggressive against women. Scientific literature has published one of the classifications of types of masculinities: Dominant Traditional Masculinity, Oppressed Traditional Masculinity and New Alternative Masculinities [4,5,6]. This article is focused on Traditional Dominant Masculinities because it is that kind of masculinity which has generated the aggressions towards the three participants of this study. Scientific research has shown that gender is a social construct [7] and other authors have evidenced how hegemonic masculinity models are associated with the use of power and violence [8]. However, there are other perspectives on this issue as well as critiques [9] that allow other analysis approaches regarding masculinities.

Even though support has been evidenced as a key factor in the recovery of women who have suffered gender violence, scientific research has identified that many women do not seek help because they fear their environment may blame them or may support the aggressor instead as well as fear of negative effects on the victim’s supporters [10]. Taking into account that gender violence often occurs in the presence of other individuals [11] many interventions to tackle this violence have focused on bystander intervention [12]. However, citizens provide us with relevant information about why it is easier or more difficult for them to support victims. The first quantitative study about this issue with 1541 participants over 18 years old identified that 40% of them had not helped a case of gender-based violence they had witnessed or had been told about for fear of the consequences they might have suffered [13].

A recent narrative study has outlined the violence exerted by harassers against those who support their victims [14]. IGV refers to the attacks and retaliation launched against gender violence victims’ supporters so that victims remain isolated. This is violence exerted both against those who help the victims and to the direct victims in order to isolate them and guarantee impunity [15]. In December 2020, the Parliament of Catalonia unanimously passed the world’s first legislation on the concept of IGV; in 2021, several parliaments are developing their own legislation [15]. IGV provokes suffering to advocates with health consequences that are being analyzed by ongoing research; but the research we present in this article is the first one in the world about the health consequences of IGV for direct victims of gender-based violence.

The physical and psychological health consequences of other types of gender violence such as sexual abuse and harassment have been studied for decades. Regarding mental health, sexual harassment is linked to suffering depressive symptoms [16,17,18,19,20,21,22,23], anxiety [20] as well as feelings of shame, humiliation and self-blame [17,18]. Furthermore, an association between sexual harassment in the workplace and suicide and suicide attempts has been found [24]. Moreover, other research carried out in different workplace settings has shown that women who suffer sexual harassment develop psychological distress, which refers to a psychological condition that is characterized by negative thoughts and feelings related to anxiety, fear or depression [25] and need later psychotherapeutic or pharmacologic treatment [23]. In addition, consequences for job-related aspects such as stress, burnout [20] and job withdrawal [22] are associated with sexual harassment. In relation to physical health, research has detected consequences related to gastrointestinal tract, loss of appetite, nausea, gastritis, stomachache [20], headache, fatigue and menstrual disorders [22].

Sexual harassment is also present on campus and colleges at universities. Sexual harassment in universities is present in the form of physical or psychological violence, sexual aggression, coercion to maintain sexual relationships, unwanted sexual attention and bribes or threats to maintain romantic relationships, among others and can be perpetrated by men such as students, professors and other staff members [26,27]. Many students who have been sexually harassed perceive permissive environments and a lack of support by the institution [28]. A qualitative analysis based on narratives identified how, in a case of sexual harassment by a male student against two female students in a master’s program, the only person who took a determined stand against the aggressor was a male professor. This study identified this upstander position within the framework of the new alternative masculinities that has always taken a stand against gender-based violence by supporting the victims [29]. Gender violence is maintained in the context of university due to the relationships created by the structure of academia, where power hierarchies and abuse are present, besides the hostility against victims, the naturalization of violence and sexist stereotypes [26,30]. Suffering sexual harassment at university affects victims’ productivity and educational performance because rejecting some teacher’s proposals implies retaliation in qualifications [31]. On the psychological level, embarrassment has been also found in victims of sexual harassment [27].

Within the types of sexual violence as gender-based violence is child sexual abuse. The long-term health consequences for those who suffered sexual abuse during childhood and adolescence have also been studied. The term used by the scientific community and international organizations is child sexual abuse (CSA hereinafter). CSA clearly refers to the fact that another person is subjecting the child or a minor who has not reached the age of sexual consent to abuse. CSA does not require any element of exchange and can occur for the mere purpose of sexual gratification of the person committing the act. Such abuse may be perpetrated without explicit force, with other elements, such as authority, power or manipulation, being determinative. CSA is a broad category that, in essence, defines the harm caused to children by forcing or coercing them to engage in sexual activity, whether or not they are aware of what is happening [32].

Research that has compared individuals with and without a history of CSA has highlighted more long-term physical health problems for those who have suffered abuse. Some of these health consequences are poorer general health, gastrointestinal, gynecological, reproductive problems and chronic pelvic pain for women, pain, cardiopulmonary symptoms such as chest pain, shortness of breath, irregular heart-beat and ischemic heart disease. Given the breadth of long-term health problems that have been linked to CSA, research has examined the wide range of psychosomatic mechanisms, finding that severe traumatic stress in childhood and adolescence can cause alterations and dysregulation in the neuroendocrine and sympathetic nervous systems that impact other body systems leading to physical problems which may not become evident until adulthood; behavioral risk factors have also been identified as being more common in adults who have been victims of CSA such as substance use, smoking, risky sexual behavior and lack of regular exercise, as well as suffering from more psychopathologies including depression and post-traumatic stress disorder [33]. Another literature review has identified, in addition to the aforementioned health problems, other health consequences such as autoimmune disorders (irritable bowel syndrome, asthma, fibromyalgia) and eating disorders in adult survivors of CSA [34]. Other research has found, among adults who suffered CSA, a perception of less social support and a higher everyday stress [35]. CSA victims are girls and boys, having serious long-term consequences for both [36,37]. Phenomenological research has analyzed the specific long-term health and well-being consequences for 7 women abused by men close to their families. All the women described a great deal of repressed and silent suffering with negative consequences for physical, psychological, relational and sexual health. The 7 women reported that the abuse continued to affect them and their loved ones [38].

However, what has not been studied is how these consequences on the health of gender violence survivors are aggravated and persist because of IGV, when attacks and retaliation launched against gender violence victims’ supporters are prolonged over time. Therefore, the aim of the present study is to explore the health and well-being consequences IGV has on gender violence survivors, that is, how seeing their supporters attacked affects to the survivors’ well-being. This article presents, for the first time, a qualitative analysis of the consequences of IGV on health and well-being of gender violence survivors. Three cases have been analyzed: two of them of sexual harassment in the university setting perpetrated by men, a professor and a student, and the other one of sexual abuse during adolescence committed by a man close to the girl family. The findings of this article contribute, first, to a more focused diagnosis so that the physical and psychological health consequences of IGV for survivors of gender violence can be better treated since the origin of the harm is not the abuse or harassment they suffered but the damage suffered by the people who support them; and second, because IGV is a type of violence that is increasingly being included in the regional and national legislations of more countries and must be diagnosed in order to quantify the damage caused.

## 2. Materials and Methods

### 2.1. Data Collection

The qualitative study is based on the analysis of the three cases. Through narrative interviews with the three women survivors of sexual harassment and sexual abuse it was possible to create a detailed reconstruction of specific experiences within a relational context and to organize these experiences [39,40]. The use of this qualitative methodology, different to the quantitative methodologies in sexual victimization, allows for capturing contextual features, defining new topics and identifying unique aspects of specific life experiences, presenting the complexities of social interactions [41,42].

Narrative interviews have been developed under a communicative approach [43] that has proven its transformative potential in the field of gender violence and sexual harassment [44]. In the dialogue that is established between researcher and participant, knowledge is jointly created through intersubjectivity and reflexivity, placing special emphasis on the interactions and social dimensions that provoke, in this case, the health and well-being consequences of IGV for the gender violence survivors. The communicative approach breaks with the unequal methodological relationship between the researcher and the person being studied, reaching a mutual consensus on the interpretation of reality.

The data for this study has been collected through narrative interviews with each participant. Interviews lasted from 15 to 40 min. Due to the current sanitary crisis, interviews were carried through videoconference. Following the postulates of communicative methodology [43], no scripts were composed for the interview, as an egalitarian dialogue between researchers’ scientific knowledge and participants’ knowledge from their lifeworld is searched. In the communicative methodology of research, no script is made. Instead, an objective is established of what is wanted to acquire information about or reflect on, and from the beginning, the objective is shared with the participant.

As a result of this dialogue, the interviews were focused on two main themes: (1) The health consequences the sexual abuse or sexual harassment had for them and (2) the consequences that seeing their supporters attacked had on them. The two topics in which the interviews were focused were chosen based on scientific literature and the exploration of the consequences that had not yet been studied of IGV on gender-based violence survivors. Both authors conducted the interviews together. In addition, the recordings of the interviews have been seen and analyzed by both.

### 2.2. Participants

Participants of this study were three women, of which two of them suffered sexual harassment in university and the other one is a sexual abuse survivor in her adolescence. Purposive sampling has been used to select participants that could answer the research objective. The three cases selected were known to the authors prior to the development of this research; because of the authors’ experience in research on gender violence in the university context. The main criterion for the selection of participants is that the people who supported them suffered IGV, i.e., the supporters were attacked and retaliated by the offender and the people who took the side of the perpetrator with the aim of destroying those supports and isolating the victims. The three cases have different characteristics and occur in different settings. Evelyn is a survivor of sexual abuse by a man close to her family, Nora suffered sexual harassment by a university professor and Chloe was sexually harassed by a male peer when they were both students in the same master’s degree. The two cases of sexual harassment were in the same university and the professors and researchers who supported them and suffered IGV are the same. In the case of Evelyn, her mother and brother supported her and suffered defamation from the abuser.

### 2.3. The Three Cases

Case 1. Woman, 40 years old. Evelyn was sexually abused from the age of 16 to 19 by the 34-year-old teacher who was giving her individual mathematics lessons. The lessons and abuse took place in the teacher’s home. The teacher gradually gained the trust of Evelyn’s family until he was considered a close member of it. He was a frequent guest at family meals and get-togethers. Soon after the first classes, the teacher began to ask Evelyn questions about her family life, friends and relationships, as well as making eye contact with comments about her beauty. Evelyn felt very uncomfortable with these situations from the beginning, but she was paralyzed. He was the teacher the whole family trusted. These types of pre-abuse interactions are known as grooming and have been extensively studied [45,46]. The first sexual abuse occurred under the pretext that the teacher invited Evelyn to his house for tea to celebrate her good marks at the end of the academic year. The abuser spun a spider’s web in which he made the victim believe that she was indispensable to him, that she had been the guilty party and that they had to keep it a secret. The abuser developed total emotional, psychological and social control over the victim for three years until she decided to break the silence by explaining it to her mother and brother. They supported her and explained the case to other close family members who also supported her. The abuser spread defamations about Evelyn, her mother and brother among her circle of family and friends but ultimately failed to achieve what he intended, which was to isolate the victim.

Case 2. Woman, 32 years old. Nora was a brilliant student who finished her undergraduate with the Distinction Award for being the best student of her cohort. The professor, whom she reported for sexual harassment, held the highest academic position at the university and had many connections with important lobbies of political and economic power. She was sexually harassed by this professor since her first year at the university. The harassment took place through emails with harassing content. The professor would also talk to her at the end of class or in the hallways. The comments soon turned to her physical appearance. In the professor’s e-mails, he included propositions to meet outside class, unwanted sexual attention (such as comments on her appearance) and abuse of power. The emails indicated that the professor could open or close doors to her academic career depending on whether she would accept his propositions or not. She saw herself forced to take a coffee with him. The determination in the answers she gave to the professor stopped the harassment in that first momentum. She soon realized (e.g., when she told a teacher) that other students and professors with important positions at the university knew that this professor sexually harassed several students every academic year for more than three decades. However, there was a deliberate silence and very few people in a position of power were willing to break it. Four years later, when she started her master’s degree, she again received another email from him, referring to her good performance in the subject he was teaching, to her potential academic future if he advised her and inviting her to have a coffee; again, showing his power relationship over her. At that moment, in shock, she asked another professor for help. Nora did not dare to report the case and asked this supportive professor to fill the complaint on her behalf, and so, he did it.

The complaint was sent to the Dean of the Faculty. She had to send it to the Equality Commission for processing. In the beginning, there was no response, but thanks to international support, the investigation process was opened. A protocol against harassment in the faculty was also approved (the first in the entire university and one of the first in the country). After the complaint, Nora’s peers at the university started to ignore her, and some professors started to publicly criticize and blame her. At the same time, she ended up obtaining the Distinction Award for her master and a competitive fellowship from the government for conducting her Ph.D. The process reached the Prosecutor’s Office, which elaborated a report in favor of the victims, recognizing on the one hand that the harassment had existed, and on the other hand, including a quote from the Dean in which she acknowledged being aware of the harassment by this professor since 1987 when she was a student.

A few years later, when Nora submitted her dissertation on sexual harassment in university, although she had conducted three research stays in the best universities of the world researching this topic, some professors (the Dean of that moment and the President of the Equality Commission) impeded her dissertation being approved twice and required changes which finally led to the approval of her PhD (outstanding valued as Excellent Cum Laude). The student assembly organized several relevant mobilizations. They also elaborated a letter against the incorporation of the denounced professor to the classes again, which was only signed by 18% of the professors of the department. All individuals that supported her suffered IGV in the form of defamation, false rumors about their private lives, criticism, threats, stalking, attacks against their children and denial of job positions for which they were qualified.

Case 3. Woman, 30 years old. Chloe was harassed by a classmate when she was studying for her Master’s degree at the university. This peer would stalk her, follow her to the bathroom or to the subway, make drawings of her naked, intimidating. She was not the only one who was being harassed, at least two peers were harassed, and other peers and teachers were also afraid of him. When she finally decided to tell someone about it, some people from her surrounding questioned her. Her boyfriend at the time ignored her and her parents recommended her to transfer to another university to end her studies. There were also members of the Equality Unit from the Faculty that revictimized her by interrogating her with accusatory questions and also professors and peers who defamed her when she decided to move forward with the case. All these reactions discouraged her from filing a complaint against the harasser. Thanks to the support received from two professors, she became empowered and decided to break the silence against this situation. Thanks to this unconditional and solidary support, she decided to denounce the case to overcome the situation. It was the first case of peer harassment won by victims at Spanish universities. Had it not been for them, victims would not have denounced the situation, and probably she would have dropped out of the university. The people who supported her suffered IGV in the form of defamation and professional consequences.

### 2.4. Data Analysis

Narratives were recorded and fully transcribed. Data were analyzed by both researchers following the communicative approach which focuses on the identification of exclusionary and transformative elements [47]. Exclusionary elements are those barriers that reproduce IGV and, consequently, its effects on the health and well-being of gender-based violence survivors and those who supported them, while the transformative ones are those which overcome them. The exclusionary data were organized into two categories: the health and well-being consequences of the sexual harassment or abuse on victims, and the health and well-being consequences of IGV on them. Moreover, transformative elements such as the effects on health and well-being of the help they received were categorized (see Table 1). The results of the narrative analysis were sent to the three participants. They gave feedback and their suggestions were introduced in the final draft.

### 2.5. Ethics

The researchers ensured that ethical standards were strictly followed throughout the research process. Informed consent was obtained from all participants, who were informed about the purpose of the study, how the information would be used, their anonymity and their right to withdraw from the research if they wished to do so. The results of the study have previously been shared with the participants who have agreed to their publication.

The study was conducted according to the guidelines of the Declaration of Helsinki and approved by the Community of Research on Excellence for All Ethical Committee with the reference number 20210708.

## 3. Results

All three participants reported health and well-being consequences when asked about the effects of the sexual harassment or abuse itself, as well as the effects of the IGV on their health. On the one hand, the sexual harassment or abuse and latter revictimization caused them different sequels such as depression, anxiety, intestinal pain, insomnia, lack of concentration and adjustment disorder. However, the three participants reflected that seeing their supporters suffer has been even more painful than the consequences of the sexual harassment or abuse itself, including fear, anxiety and insomnia.

### 3.1. Health and Well-Being Consequences of the Sexual Harassment or Abuse and Revictimization

The most common health and well-being consequences in the three survivors are anxiety, constant nervousness, intestinal pain, insomnia and fear. For Evelyn, the abuse caused her intestinal pain, depression, stress, anxiety and panic. However, intestinal pain disappeared when she told her mother and brother about the abuse:

When the direct abuse happened, it is very clear that I had chronic intestinal pain, which I had every day. That pain disappeared when I decided to explain for the first time what was happening and what had happened. Also depression, stress, anxiety, panic, moments of panic. (…) Some things are common, but the intestinal pain and panic are very related to that moment.

(Evelyn, interview)

In the case of Evelyn, she was also diagnosed with adjustment disorder.

Stress and anxiety are constant in all the revictimization period, but, in addition, I am diagnosed with adjustment disorder, that is a kind of disorder that people who have experienced a very strong trauma have. This disorder can disappear at the moment when this does not happen anymore when the thing is not there anymore.

(Evelyn, interview)

Chloe felt stomachache and explained that the constant nervousness she felt due to the sexual harassment caused all the food to make her sick. In addition, she also suffered great and constant fear and insomnia. In fact, these consequences of the harassment changed her everyday life.

It created a feeling of fear on me, a lot of fear. I had never felt so vulnerable and insecure to just walk and do regular things we all do everyday. I even had to ask for help to go from the metro station to home (…) Stomachache, due to a lot of nervousness, eating and feeling sick because of the food, I imagine it was because of the nervousness. (…) I remember one time I was walking through campus and the harasser was walking behind me. And I felt like my legs started to shake. I felt like I didn’t control the situation at all.

(Chloe, interview)

For Nora, the most harmful health consequences she had were related to the revictimization she suffered after filing a complaint against her harasser. She felt constant nervousness, anxiety and fear, as well as insomnia. Her doctor, a medical practitioner, gave her antidepressants because of the situation.

I would relate it to constant nervousness. I remember in the beginning, after the complaint, seeing many changes in my surroundings. That suddenly people treat you in a different way. The same people. They stop talking to you, they stop saying ‘hi’, like something very subtle but every day something happens. I didn’t expect that. On a psychological and mental level … being a normal person who has friends (…) suddenly you realize you are weird, like a weirdo who has done something. (…) The lack of sleep is also explained by this because you don’t understand what’s happening.

(Nora, interview)

In the case of Chloe, revictimization and having some people not supporting her caused her to cry and feel uncomprehend.

The situation overwhelmed me too much and I didn’t know what to do to fix it. I felt very insecure in my surroundings because I had supporters, but I also had people who did not support me (…) that made me want to cry and feel a very big incomprehension.

(Chloe, interview)

Another consequence reported in the interviews is related to the performance in the job and academic careers of participants. The three participants experienced difficulties concentrating due to the sexual harassment, abuse and revictimization.

At work, related to health, I also remember the lack of concentration, because you can’t focus.

(Nora, interview)

In addition, for Nora, the criticism she had to face by her professors and other people because of having filed a complaint about the harassment, made her academic self-esteem lower despite the fact that she had always been a brilliant student. Those professors who questioned her were not her supporters. On the contrary, they were people who took sides with the harasser.

You face many criticisms: what must have you done, as if you had provoked the harassment. They criticized my work in class because they said I wasn’t doing it right. I was puzzled and it literally took the sleep out of me because the same teachers that valued me so much before, the same ones, started to criticize me when I became a victim. (…) Being criticized in public creates a big hang up and a feeling of inferiority. They say you are no longer a good student. (…) It is not a matter of pride; it is that you end up believing them and you say: maybe I don’t do it so well (…) You feel sensitive but also very vulnerable.

(Nora, interview)

### 3.2. Health and Well-Being Consequences of IGV on Gender Violence Survivors

The three participants explained that seeing their supporters suffer provoked suffering in them, even more than the consequences of the sexual harassment or abuse. Now the health and well-being consequences were of a different origin but manifested themselves by reliving some of the symptoms they had suffered from sexual harassment or abuse or other symptoms. Nora saw how the male professor who first supported and reported for her, as well as other women professors and researchers that supported her, were suffering IGV that even affected their children, job status and reputation. Knowing their harm made Nora feel anxious, and even made her wonder who to tell her situation because she knew supporting her could have great consequences:

You feel bad, seeing that those people are going through a bad time because of you, because of protecting you, it affected my health. You may think that it would be better not to tell someone because somehow you are forcing them to support you because you can’t go on without support. So you have no one to call. You call the same people. That generates incredible anxiety.

(Nora, interview)

In the case of Evelyn, seeing the defamation against her supporters made her feel again all the pain she had gone through when the abuse happened. In fact, the disorder she had as a consequence of the abuse and had disappeared when she told people about her abuse, came back again. These attacks also generated stress, anxiety, lack of concentration and insomnia.

When I perceived that people from my family or from outside that had supported me were being attacked, I felt everything again. The [adjustment] disorder appeared again. It is a constant struggle. (…) This constantly gives you stress and anxiety. These two come together with the other one. And well, it also provokes lack of concentration, because you are mentally and emotionally bad … it is difficult to concentrate and you scatter. (…) This also derives into chronic tiredness in most stressful periods. Everything is connected, these effects are connected, and it also gives you insomnia, evidently.

(Evelyn, interview)

Chloe, in the beginning, felt great relief and hope when some people from her university started supporting her. However, when she learned that her supporters were being attacked, she also had anxiety, nervousness and distress as well as a feeling of guilt because she thought supporting her was what made those people suffer.

The fact of starting to see that the attacks were not only against me when I wanted to file a complaint, but also were against those people gave me a very strong feeling of anxiety and anguish, because I thought … I even felt guilty: these people are suffering because of a situation that has happened to me.

(Chloe, interview)

Nora explained in her interview how she had outbursts whenever she heard the defamations carried out against her supporters. She broke into tears or felt anxious when hearing them or when seeing people who made up those defamations. As an example, she explained how some friends of hers once made a joke about those defamations trying to play the matter down. However, she broke into tears when she heard the joke.

But they said it jokingly, they were people who didn’t want to harm me, they wanted me to be alright, they were like: that [defamation] is ridiculous, let’s just laugh about it. And I remember I broke into tears at that very moment because I didn’t find it funny. And then I thought: ‘you are not OK’.

(Nora, interview)

Seeing IGV carried out against their supporters was even more painful for the three participants than the sexual harassment and abuse they suffered. The three of them agree that they would rather receive the consequences for themselves than seeing their supporters being harmed.

I think you suffer more for the other people and you have more health consequences because of worrying about him than for me. For him and for the whole investigation center that supported me. (…) They still say something, and I start crying because of what it has meant to all of them. (…) This was continuous and the suffering of a lot of people. So, the suffering is bigger than a thing that has just happened to you, it is obvious. (…) Do you think I cared about my master’s project? I mean, I worried about getting good grades, but I mean, I would have run away from there and done something else.

(Nora, interview)

At times, I’d thought I’d rather suffer [the attacks] on me than seeing other people affected. And that generated a lot of nervousness, anguish, and anxiety. I didn’t fully understand why other people had to go through what I was going through if the harassment only happened to me.

(Chloe, interview)

Evelyn also said that the most painful feeling was seeing her supporters harmed, and that it had greater health consequences on her than a direct attack on her. When they broke the silence about the abuse and told the rest of the family about it, seeing that the abuser defamed and spread lies about her mother and brother was devastating for her.

This has generated a more negative impact because you love the people who support you and to me, it hurt more than they were being harmed than receiving a direct attack on me. I have it very clear, and that is why the disorder develops more … the adjustment disorder and all that. (…) What you suffer as a victim is not only the harm done to you, but the harm is done to your supporters, it duplicates the effect it has on your health seeing them suffer too. That is terrible when you feel it, the effects you have as a victim are multiplied.

(Evelyn, interview)

### 3.3. Positive Effects on Survivors of the Support Received

However, all participants emphasized that despite the suffering and consequences of IGV had on them, the support they received contributed to their recovery. All of them agreed that, without the support, they would not have made it to where they are today. They would not be survivors. The three women are involved in the fight against gender-based violence and are successful in their life and work. For example, Nora is now a recognized researcher in the field of law and sexual harassment. She thinks she would not have pursued her career at university if it had not been for the support she received. This support also reduced the health consequences of the sexual harassment she suffered, such as dealing better with the fear.

The support for me was the key, but I don’t say it because it is a beautiful phrase. I wouldn’t have been here, but I wouldn’t have finished my master’s and PhD, I’m telling you. (…) I have had fewer consequences and I have been much better because of the support. To have changed from victim to survivor is very meaningful because you survive the situation and end up smiling. Somehow, you win. There are things that are still there and they will always be there, but you know how to deal with the fear better.

(Nora, interview)

Chloe explained that many health consequences the sexual harassment had caused disappeared when she received the support. Seeing people who cared and took a stand against the harassment she was suffering empowered her to live her everyday life with less fear. Chloe, together with Nora and others, have created a solidarity network to support victims of gender-based violence at the university.

The issue of my legs shaking, I remember very clearly how it marked a before and after. The moment I felt I had support and that people were thinking about me and protecting me, I remember going through the corridor and I thought: ‘Chloe, your legs can’t shake anymore’. Because I felt supported. It happened the same with many other things, issues that went away, many fears that I had, disappeared when I had more support. I went from a very vulnerable situation where I wanted to run away from the university to file a complaint against the harassment. (…) It was 100% thanks to the people who took a stand in a very clear way.

(Chloe, interview)

For Evelyn, the support received has also been essential. She explained that, although all the health consequences have not disappeared, they have been diminished thanks to the support. In addition, she declared that those health consequences are still happening because her supporters are still receiving IGV. However, she thinks that without her supporters she would have ended up in a very bad situation. In fact, she states that the support received has saved her life and that seeing people who truly work against sexual abuse has made her want to be well so she can help other victims and put an end to this issue. She is now a media professional known for her excellent coverage of news related to gender-based violence and sexual abuse.

Without it, I would be under a bridge, it is like that. Receiving the support saved me. The consequences don’t disappear, but they are mitigated. Without the support it is impossible, you end up in… I could perfectly be an intern in a psychiatric hospital. Or I could not be alive, it is so hard that without the support I would be either not alive or in a psychiatric hospital or I would have developed a much worse mental illness and would be dead in life. (…) When you see that they really support you and believe you, it has a direct impact on you. And when you see there is real work to end up with this filth, it fills you with meaning and helps you fight every day. If you are not alright, you will not be able to help, and I have it present every day of my life.

(Evelyn, interview)

## 4. Discussion

The negative health and well-being outcomes of sexual harassment or abuse and revictimization as gender violence have been widely investigated [16,17,37,48]. However, it is the first time the health consequences of IGV on gender violence victims are being studied. Overall, the qualitative findings of these three cases show that IGV not only has harmful effects on these three victims’ advocates but also on these victims of gender violence. Apart from trying to isolate these victims by attacking their supporters [14], seeing them suffer has caused negative health and well-being consequences in these three participants, even more than the sexual harassment or abuse itself. That is, what has caused the harm in the studied cases has been the attacks the harassers have made against the people that supported them. However, the three victims emphasized that the support received was crucial to overcome the oppression they were going through.

First, the two participants who suffered sexual harassment have reported negative health and well-being outcomes caused by the sexual harassment, such as fear, anxiety, depressive symptoms, stomach ache, lack of appetite and insomnia, which are consistent with previous literature [16,17,18,19,20,21,22,23]. Evelyn due to the sexual abuse, experienced intestinal pain, adjustment disorder, anxiety, depression, insomnia, panic and chronic stress, which has also been highlighted by research as CSA health and well-being consequences [33,38].

Second, seeing the attacks against the ones that supported them and helped them overcome the situation has been, according to the three participants, even worse than suffering the sexual harassment or abuse itself. In fact, for Evelyn, the attacks against her supporters replicated the health consequences of the sexual abuse she previously had. For Nora, the IGV her supporters suffered made the constant nervousness and anxiety she already had due to the sexual harassment and revictimization persistent. Finally, Chloe had great anxiety and anguish when she knew that the people supporting her were suffering. Thus, although some research has evidenced the risks of intervening in gender violence cases [49], the health consequences of IGV on gender violence victims should also be addressed. Participants have also emphasized that some of these health and well-being consequences are still happening to them because the attacks against their supporters have not ceased. In addition, feelings of self-blame were also very present in the three of them, as they reported that, sometimes, they would rather have suffered the consequences alone without involving anyone else.

In spite of the negative health and well-being consequences of IGV on participants of the study, the three of them emphasized that the overall outcome of the situation they suffered had been more positive because of the support received. They were able to cope much better and became survivors thanks to the support they received. In regard to Nora and Chloe, they stated that they would have left their career in academia had they not received the support. In addition, the health consequences directly related to sexual harassment were mitigated in the three cases due to the support. Nora and Chloe lost many of the fears the harassment had generated on them. Evelyn stated that the support had saved her life. In this vein, scientific literature has already shown how receiving support helps the recovery of the survivors and is essential to cope with the sexual coercion suffered in college [26,50]. In conclusion, the three participants acknowledged that the support they received helped them overcome the harassment or abuse, although they had suffered while seeing their supporters being attacked by the harassers.

These findings shed light on a reflection that must be made on support to gender violence victims. In this study, participants have had a better global result due to the support received, but there can be situations in which the global result is worse because the negative health consequences of IGV on gender violence victims are bigger than the positive ones. Thus, the global outcome consequences of the support must be estimated in order to guarantee better health for gender violence victims.

As an example, research has evidenced that some women victims of Intimate Partner Violence prefer their children not to become involved, so they discourage them from defending them in front of the batterer. They do so because they are afraid of their children’s wellbeing [51]. Literature has stated that what children do in the context of domestic violence also has an influence on their mother’s well-being [52]. Mothers victims of domestic abuse have emphasized that their children’s well-being translates into their well-being and seeing their children suffer has created self-blame [53,54]. In these cases, receiving this support and seeing IGV against their children would have more negative consequences on them. In the present study, participants reflected on who to tell or not because they knew how hard the consequences of IGV were for their supporters.

The findings of this study show that the three participants had somatization caused by seeing IGV affect their supporters, that is, they had health and well-being consequences for something that did not directly happen to them. It must be taken into account that the source of the pain is different and, therefore, the treatment and therapy needed to overcome it is different. Following the previously mentioned example, scientific research demands that, although many programs have focused on the recovery of the mother in domestic violence cases, child-centered interventions are also necessary for ensuring the well-being of both [52].

Feelings of self-blame are common in cases of gender violence and victimization [18]. In this study, the three victims had feelings of self-blame because of IGV, because of feeling that they had caused suffering to their supporters. In the case of not receiving any help, the solution lies in overcoming psychological consequences of the revictimization [55]. Nevertheless, when the source of the self-blame is the suffering of their supporters, it may be necessary for the supporters to overcome the situation.

The study’s findings have new and important implications. Public health must take into account IGV to offer a better response to gender violence victims. Practitioners from the fields of psychology and psychiatry should take into account that IGV could cause somatization such as on the three gender violence victims’ cases studies, in order to analyze causes of physical or psychological effects, diagnose more accurately and provide better care. Authorities should also be informed of the possible consequences of IGV on gender violence victims to develop public policies that prevent this potential social and health issue.

## 5. Conclusions

The study of these three cases shows that IGV has not only negative consequences on the supporters of these gender violence victims, but it also negatively affects the direct victims themselves. Thus, addressing IGV is not only beneficial for IGV victims, but it is also positive for gender violence survivors’ health and well-being.

While it is true that participants report that the IGV suffered by those who supported them has caused them more pain than the sexual harassment or abuse, they also report that without that support they would not have been able to overcome the situation and the consequences for their physical and psychological health and well-being would have been much worse.

A limitation of the study is that it is based on only three narratives. More qualitative and quantitative research is needed to obtain consistent findings on the relationship of IGV and the health and well-being of victims of gender violence. Nevertheless, having three participants has been a choice that has enabled to deepen more into their narratives and acquire a more profound knowledge of the consequences IGV has had on them. In qualitative studies, the results are consolidated when similar results are obtained in other contexts. This research is pioneer because it analyzes for the first time the consequences of IGV on gender violence survivors’ health and well-being. However, the results regarding health and well-being outcomes of sexual harassment and abuse are consistent with other studies. Future quantitative research should analyze the consequences of IGV on gender violence survivors in order to know the extent of the suffering caused and to be able to assess the global outcome of the situation with the help received. More qualitative research could explore in depth effective ways of helping gender violence victims for their global well-being outcome to be positive. Future research should also focus on developing tools to provide a better diagnosis and treatment of IGV in both IGV victims and gender violence victims.

## Figures and Tables

**Table 1:** Summary of the elements and the categories related to them.

Elements	Categories
Exclusionary elements	1. Health and well-being consequences of gender violence 1.1 Psychological health consequences 1.2 Physical health consequences
	2. Health and well-being consequences of IGV on survivors 2.1 Psychological health consequences of IGV 2.2 Physical health consequences of IGV
Transformative elements	3. Outcomes of the support received 3.1 Process of formal complaint 3.2 Mitigation of consequences

## Data Availability

The narrative interviews were recorded and transcribed in their entirety. These documents remain anonymized and archived in a secure location to which only the researchers have access to ensure the confidentiality and privacy of the participants.
